# Editorial

**Published:** 1900-02-15

**Authors:** 


### EDITORIAL.

The difficulty in diagnosing obscure diseases associated with the teeth has been a great drawback to the advancement of dental therapeutics as a specialty of dental practice. There is good reason to believe that many curable teeth have been sacrificed by the dental surgeon which a skilful therapeutist would have saved, had he been able to determine with definiteness the character and extent of the diseased area. We have been accustomed to depend for our diagnosis of hidden lesions about the teeth upon the history of the case and such ocular and tactile impressions as we have been able to secure. More recently the electric light has come to our aid, and very gratifying results have been had by illuminating the parts suspected, and, by observing the contrasts of shadows, translucency, etc., we have been able to fortify our guesses and secure tolerably accurate views of deeply hidden structures. But now comes the Roentgen ray, which throws an entirely new light on this dark problem, and we hail its promised benefits with much satisfaction. The article by Dr. Price in this number, and the article by Dr. Kells in the October (1899) *Dental Cosmos*, will be well worth considering. The results shown justify us in hoping that at last we have a method of making plain the hidden things, and when this can be done it will not take the average practitioner long to devise ways and means for removing the threatening environment and giving the needed aid for a restoration to normal conditions.

The great obstacle now seems to be to provide a satisfactory apparatus, and at moderate cost; but this no doubt will come as soon as its need is fully demonstrated and the profession has come to appreciate its value to the extent of stimulating manufacturers to supply the demand.

The physiology of the dental tissues, more particularly of the dentin, has received much attention, but is no more definitely settled, perhaps, than in the days of Hunter and Bell, and probably it will still remain a puzzle to dental scientists until the histology of the pulp and dental fibrillæ is more satisfactorily made out than at present. We are therefore privileged to theorize as much as our fancy pleases, so long as we keep our theory well within logical, if not scientific, bounds. The article of Dr. Junkerman on the hydro-mechanical theory of sensibility of dentin is very interesting and, although an old theory, it is remarkably well stated and furnishes much food for thought and careful consideration. With our present knowledge the hydro-mechanical theory will serve as well as any other upon which to base practice. It is to be hoped, however, that we may soon be able to do more than theorize about a matter which is of so much consequence. Our methods of obtunding sensitive dentin are the result too largely of clinical experimentation, and necessarily this must be so until we comprehend the vital functions of this structure. Just how far we are justified in estimating the character of this function, from our experience in handling it, is questionable, at least from the scientific standpoint. What conclusion, for instance, may we draw from the oft-repeated statement that sharp engine burs, run at high speed by some and low speed by others, offer the most practical method of excavating carious cavities without pain? And likewise, if we were to analyze other well known methods, we should find it difficult to harmonize the results sufficiently to entitle us to dogmatize as to the nature of the tissues affected. Will not our biologists, physicists, or vitalists if you please, take hold of this important matter and let us have the truth about it?

